# The Relationship Among Financial Inclusion, Non-Performing Loans, and Economic Growth: Insights From OECD Countries

**DOI:** 10.3389/fpsyg.2022.939426

**Published:** 2022-07-04

**Authors:** Peng Zhang, Mengxue Zhang, Qiancheng Zhou, Syed Anees Haider Zaidi

**Affiliations:** ^1^School of Humanities and Social Sciences, Anhui University of Science and Technology, Huainan, China; ^2^Department of Management Sciences, COMSATS University of Islamabad, Sahiwal, Pakistan

**Keywords:** financial inclusion, non-performing loans (NPL), economic growth, Organization for Economic Corporation and Development (OECD), Driscoll-Kraay standard errors

## Abstract

This study implies finding the linkages among financial inclusion, non-performing loans (NPLs), and economic growth. The study uses large panel data of 21 Organization for Economic Corporation and Development (OECD) countries for the dynamic panel estimation by using the Driscoll-Kraay standard errors with fixed effect. The results of the dynamic panel estimation technique revealed the existence of a long-run relationship among financial inclusion, NPLs, and economic growth. Financial inclusion contributes positively to economic growth by reducing NPLs. Furthermore, NPLs negatively impact financial inclusion as well as economic growth. The study presents important policy recommendations to control NPLs and boost the level of financial inclusion in the selected economies.

## Introduction

Poverty is considered the utmost challenge to achieving global sustainable development. “No Poverty” is set to be the foremost goal in the 17 Sustainable Development Goals (SGDs) set by the United Nations (2015). The World Bank road map also attains Universal Financial Access by 2020 to reduce poverty and enhance financial inclusion to the maximum level (WEF, 2017). Extensive empirical research has highlighted the relationship among financial inclusion, growth, and poverty. Financial inclusion means providing affordable financial products and services (like insurance, savings, transactions, credit, and payments) to the deprived sects and individuals of society. It is learned that the access and availability of financial resources are directly linked to poverty reduction (WEF, 2017). Demirguc-Kunt and Klapper (2012) analyzed the global financial inclusion (GFI) database (Global Findex) of 148 economies for investments, credits, payment systems, and risk management systems. The research found that about 50% of adults have formal accounts in financial institutions worldwide. In other words, half of the world's adult population is not included in the formal financial sector.

This study primarily deals with financial inclusion, concerning the assessment and obtainability of formal financial services, like bank deposits, insurance, and credits for all financial stakeholders of the economy. The issue of poverty and low financial inclusion go side by side, which is pertinent in the case of developing countries. The degree of high financial inclusivity entails the environment and circumstances in which the majority of stakeholders are using formal financial systems and services with the stability of capital. This requires monetary easing, a robust legal policy, a low cost of financial services, a variety of financial products, and comfortable and closer access to financial institutions in a country (Allen et al., 2016). As a result, the level of financial inclusion states the level of growth of a country (Beck et al., 2007). Numerous studies have validated the positive linkage of financial inclusion with economic development (Sarma and Pais, 2008; Babajide et al., 2015; Kim et al., 2018).

The willingness and ability of economic players or customers to engage with the formal financial system are one of the critical determinants of financial inclusion and economic growth. But customers' unwillingness and ability to use the formal financing sector creates a void. In this context, the concern about financial literacy remains valid. Likewise, the misuse of formal loans causes an increase in non-performing loans (NPLs), hence financial exclusion. For instance, the personal use of a business loan by a customer restrains his/her ability to repay and results in a bad or NPL. It means that NPLs coined by financial illiteracy are negatively associated with financial inclusion. So, the quality of financial framework and quality of customers are essential to attain financial development, inclusion, and economic growth (Grohmann et al., 2018). Therefore, besides the research on institutional and regulatory determinants of financial inclusion, the customer dynamics also need extended scholarly attention to attain a required level of financial inclusion.

From a broader perspective, the relationship between financial inclusion and economic growth is somehow explained by the level of NPLs in a country. It means that a weak financial system along with NPLs on the part of the household and corporate level triggers strict lending requirements and reduces the level of monetary easing, causing distance between the poor and formal financing sector. Therefore, the financial exclusion caused by NPLs seems to have direct implications on the economic growth of a country. It has been observed that different macroeconomic variables affect financial inclusion and NPLs in unique ways, but NPLs and GDP are directly associated with each other (Beck et al., 2013).

The case of Organization for Economic Corporation and Development (OECD) countries is unique in this regard. OECD is the most prominent group of countries with pioneering efforts to boost financial inclusion. For example, the establishment of an international network on financial education (INFE) in 2008, with 230 member organizations from 100 countries. Later in 2010, INFE and a Russian trust fund introduced a collaborative project to improve financial literacy and inclusion to attain financial stability in OECD countries. Yet the OECD countries recorded the highest level of NPLs, i.e., 4.121% of the total gross loans in 2009. The surpassing NPLs called up strict operational measures and reduced the loaning capacity of financial institutions denting the level of financial inclusion (IMF, 2017). Therefore, it is perceived that along with the other macroeconomic variables, financial inclusion and NPLs are significant challenges for the OECD countries. So, this study explores and presents the case of NPLs, financial inclusion, and economic growth of the OECD countries.

For this purpose, this study assesses the dynamic relationship among financial inclusion, NPLs, and GDP per capita controlled by other economic variables, i.e., population, school education, inflation, trade, urbanization, rule of law, and infrastructure. This study adds to the literature in three ways. To begin with, the studies examine the influence of financial inclusion and NPLs on economic development in the OECD nations. Second, while calculating the impact, we take both the demand and supply sides into account. People's credit requirements are on the demand side, while financial services are provided on the supply side (such as ATMs, bank deposit accounts, and life insurance). Finally, because the OECD nations are intimately linked by geography, commerce, and financial integration, we evaluate the possibility of cross-section dependence. Furthermore, the financial inclusion index namely “Findex” is used as a proxy of financial inclusion. We apply the Driscoll-Kraay standard errors with the fixed effect regression technique. The results of the dynamic panel estimation technique revealed the existence of a long-run relationship among financial inclusion, NPLs, and economic growth. Financial inclusion contributes positively to economic growth by reducing NPLs. Furthermore, NPLs negatively impact financial inclusion as well as economic growth. Table 1 reflects the detail of definitions of variables used in this study.

**Table 1:** Definitions of study variables.

**Abbreviation used**	**Variable name**	**Definition**
Findex	Financial inclusion	Index includes the proxies of financial inclusion namely ATMs, Deposits, Bank Branches and Life Insurance.
NPL	Non-performing loans (NPLs)	In banking, commercial loans are considered non-performing if the borrower is 90 days past due.
GDP	Gross Domestic product (GDP)	Its measurements include gross domestic product which is divided by population for getting Per capita GDP.
Edu	Education	It is the total new entrants in the last grade of primary education, divided by the population at the entrance age for the last grade of primary education.
Inf	Inflation	Inflation is a sustained increase in the general price level of goods and services in an economy over a period of time.
C	Corruption	It captures ability to curb the extent to which public power is exercised for private gain.
INFRA	Infrastructure	It is an index prepared by authors using PCA by including telecommunication (telephone and internet) plus energy electricity use per capita.
Trade	Trade	It represents the value of all goods and other market services provided to the rest of the world.
Pop	Population	It refers to people living in urban and rural areas as defined by the nationwide statistical offices.
RoL	Rule of Law	It captures perceptions of the degree to which agents have confidence in and abide by the rules of society.
Unemp	Unemployment	It refers to the portion of the labor force that is without work but available for and seeking employment

The sequence of the remaining part of this article is as follows: Section Review of Literature provides a brief review of literature. Section Data and Proxy Measures describes the data set and measurement of the proxy for financial inclusion and economic growth; Section Methodology reflects the methodology of the paper; and Section Conclusion includes the conclusion part of this paper.

## Review of Literature

### Financial Inclusion and Economic Growth

Since the late 1990s, there has been increasing attention to the term financial inclusion explaining access to financial services for the needy in a country (Leyshon and Thrift, 1993, 1994). The studies have explored the effect of financial inclusion on economic growth. It is known that financial inclusion has a positive relationship with economic growth. For example, Kim et al. (2018) studied the organisation of islamic cooperation (OIC) countries and found a significant positive impact of financial inclusion on economic growth. Similarly, in 11 MENA (the Middle East and North Africa) countries, Naceur and Ghazouani (2007) also reported a positive relationship between financial inclusion and economic growth. Similarly, Makina and Walle (2019) studied the case of Africa, and Cabeza-García et al. (2019) tested a sample of 91 developed and emerging economies and explored that financial inclusion significantly boosts economic growth. Furthermore, it is also believed that financial development adding to financial inclusion can reduce the level of poverty (Boukhatem, 2016). Likewise, Pradhan et al. (2016) examined the relationship between insurance penetration and economic growth for the ASEAN Regional Forum (ARF), revealing short-term co-integration and causality between insurance penetration and economic growth.

H_1_: There is a positive effect of financial inclusion on economic growth.

However, it is pertinent to note that there could be a potential effect of economic growth on financial inclusion. Based on the modern economic theory, economic activity generates chances to engage with mainstream financial institutions. This situation creates a viable opportunity to observe the effect of economic growth on financial inclusion, where literature seems silent. This study approves the effect of financial inclusion on economic growth and tends to validate the potential effect of economic growth on financial inclusion. Therefore, we postulate that economic growth may have a positive effect on financial inclusion as well. Our hypothesis is:

H_2_: There is a positive effect of economic growth on financial inclusion.

However, the relationship between NPLs and financial inclusion requires further attention. There is a need for a clear understanding of the factors contributing to financial inclusion. In this regard, the factor of NLPs remains novel.

### Non-Performing Loans and Financial Inclusion

The linkage between NPLs and financial inclusion lacks theoretical and empirical understanding. It has been established that financial inclusion may increase the magnitude of NPLs. For example, Chen et al. (2018) reported a positive impact of financial inclusion on NPLs in the context of China. Similarly, Ozili (2019) also found that an efficient and stable financial system leads to a higher NPL rate. To check this stance, we propose the following hypothesis:

H_3_: There is a negative effect of financial inclusion on NPLs.

However, the literature is silent to report the effects of NPLs on financial inclusion. On the institutional side, it is observed that credit growth has a positive relationship with NPLs, harming banking profitability, and financial inclusion (Vithessonthi, 2016). NPLs become a financial burden for banks and ultimately reduce their loaning capacity. The threat of NPLs lessens the financial inclusion because of the education and consumption behavior of borrowers.

In another view, it is considered that household and small loans intrigue risk insensitive credit extension for banks and other financial institutions. This may cause a lower ratio of NPLs and boost financial inclusion (Calomiris and Kahn, 1991; Song and Thakor, 2007). Although the literature provides ample detail about the effects of financial inclusion on NPLs, the reverse relationship has not been investigated to check the effects of NPLs on financial inclusion. Based on the types and size of loans, it can be concluded that NPLs also affect financial inclusion in the country. This study fills this gap and examines the effect of NPLs on financial inclusion with the help of the following hypothesis:

H_4_: There is a negative effect of NPLs on financial inclusion.

### Non-Performing Loans and Economic Growth

The relation between NPLs and economic growth is widely discussed in the literature. The scholarly discussion has mainly focused on determinants of NPLs, recognizing the negative association of GDP growth and the positive relation of inflation factors with respect to NPLs (Mazreku et al., 2018). Just as a stable financial system promotes economic growth, a poor financial system that struggles with NPLs and inadequate capital could undermine growth (Schumpeter, 1954). In credit modeling, Zeng (2012) views loans to the economy as increasing total demand and thus offering a significant social benefit, whereas NPLs are considered a source of financial pollution (Minsky's Approach to Poverty and Unemployment, 2015).

In modeling credit, Zeng (2012) views loans to the economy as boosting total consumption and hence yielding a positive social utility, while NPLs are viewed as a source of financial pollution (Minsky's Approach to Poverty and Unemployment, 2015; Stiglitz and Andrew, 1981) that negates social utility. Zeng identifies two economic implications of NPLs. First, if NPLs grow, economic growth could decline, causing inefficiency in the allocation of resources. Second, capital requirements will rise as a result of NPL expansion, as capital depletion occurs because funds are stuck in these institutions, making it difficult for banks to finance new, economically viable projects. The NLPs significantly affect banking performance as well as economic growth (Podpiera and Weill, 2008; Campanella et al., 2020). These studies suggested that improving the credit selection criteria avoids the hazard of NPLs. Some researchers (e.g., Podpiera and Weill, 2008; Vithessonthi, 2016) reported that overall economic conditions (i.e., economic crises and normality) could differently affect the banking credit extension and NPLs. Likewise, Partovi and Matousek (2019) stated that NPLs could cause uncertainty, economic downturn, and instability. Many studies state that NPLs are undesirable outputs that contribute to economic growth and banking inefficiency (Kumar et al., 2018; Ozili, 2019).

NPLs reduce the banks' loan granting capacity and weaken their role in economic development. The research indicates that GDP has an inverse relationship with NPLs (Badar and Yasmin Javid, 2013; Khan et al., 2015; Kjosevski and Petkovski, 2017). Louzis et al. (2012) and Endut et al. (2013) also identified macroeconomic variables such as inflation, trade, and illiteracy causing higher NPLs. In general, it is found that there is a negative relationship between NPLs and economic growth. This study testifies this relationship through the following hypothesis:

H_5_ There is a negative effect of NPLs on economic growth.

The bad quality of loans and credit extension criteria is often blamed for the increasing trend in NPLs and economic crises (Bancel and Mittoo, 2011). In this context, there are a number of studies suggesting regulatory enhancements to overcome the state of increasing NPLs (Carter and McNulty, 2005; Yang, 2017). At the same time, the world has observed the rapid success of microfinancing and its impact on banking efficiency and economic growth (Chatterjee et al., 2006; Collins et al., 2009).

The literature has largely discussed the effect of NPLs on economic growth but the vice versa effect has not been checked so far, especially for the OECD countries. The panel of the OECD countries is diverse with different income and growth levels. It will be interesting to check the effects of economic growth on NPLs for different income levels. This study contributes to highlighting such effects and proposes the following hypothesis:

H_6_ There is a negative effect of economic growth on NPLs.

The nexus of financial inclusion, NPLs, and economic growth is interesting and imperative to study. The accomplishment of sustainable development goals is not possible without reducing poverty through financial inclusion. The factors associated with financial inclusion are studied in this paper for seeking the attention of policymakers. It is a positive sign and fortune for current researchers that the data released by the World Bank regarding the financial inclusion index are available to the researchers for empirical investigations of the concept of financial inclusion and other relevant factors.

This global index includes primarily the supply side of financial inclusion. NPLs (not addressed by Global Findex) are important demand-side variables that impact financial inclusion and economic development. This research work is an attempt to find more insights into the relationship among NPLs, financial inclusion, and economic growth in the presence of macroeconomic variables.

## Data and Proxy Measures

### Structuring the Panel Dataset

This research examines the relationship among financial inclusion, NPLs, and economic growth of the OECD countries for the sample period of 2004–2017. The selected sample period for the OECD countries partially covers the timeline of the Sustainable Development Goals by the United Nation. The study considers panel data of 21 OECD countries out of 36 countries, because of the missing values and data limitations. It is argued that 58% of the OECD countries' data may provide generalizable findings. The data of related variables have been retrieved from the Global Democratic and Development Foundation (GFDD), the World Development Indicator (WDI), and the Financial Access Survey (FAS).

### Proxy Measures

Financial inclusion entails how many individuals and businesses use financial services. In this context, the variables such as account penetration, deposits, loans, and insurance are important factors in assessing the level of financial inclusion (Demirguc-Kunt and Klapper, 2012; Van der Werff et al., 2013). A study by the World Bank (2018) has used various proxies like financial institution accounts, financial institution loans, electronic means of payment, and debit card ownership on the basis of (percentage, 15+ years) to establish the index of financial inclusion.

The first proxy to calculate financial inclusion is the proxy for savings “deposit accounts with commercial banks per 1,000 adults” indicated as DEPO (Kim et al., 2018). The second proxy is automated teller machines per 100,000 adults indicated as ATM, which can be a suitable measure of the ownership of accounts. It is assumed that the ownership of accounts indicates how many people or firms have accounts at formal financial institutions. Although the best way to measure this key factor is by counting the number of people or firms who have accounts, the data of the World Bank is limited to 2011. Therefore, we use ATM as a proxy for the ownership of accounts. It is because formal financial institutions generally issue either a debit card or an ATM card tied to an account, the ATM penetration rate will indirectly represent the accounts penetration rate.

To measure the penetration rate of financial institutions, we use proxy of bank branches per 100,000 adults indicated as BRCH. This proxy may be helpful to assess the presence of financial institutions and suitable to infer the access to the financial institutions by the general public and institutions or firms. Life insurance premium volume to GDP (LINSUR) is our fourth proxy to assess the level of insurance penetration. The access to insurance institutions is used to mitigate the financial risk and preserve a person's financial stability from unpredictable accidents. Therefore, LINSUR can be an appropriate proxy to measure the level of awareness regarding risk mitigation and financial stability (Kim et al., 2018).

In addition to the IMF Findex proxy, we have also developed the Findex proxy by employing Principal Component Analysis (PCA) through the above-mentioned proxies. The related statistics of PCA are given in Table 2.

**Table 2:** Principal component analysis.

**Sample: 2004 2017**					
Included observations: 294					
Computed using: Ordinary correlations					
Extracting 4 of 4 possible components					
Eigenvalues: (Sum = 4, Average = 1)					
**Number**	**Value**	**Difference**	**Proportion**	**Cumulative value**	**Cumulative proportion**
1	2.12303	1.34408	0.5308	2.12303	0.5308
2	0.77895	0.100792	0.1947	2.90198	0.7255
3	0.678158	0.258296	0.1695	3.580138	0.895
4	0.419862	–	0.105	4	1
**Eigenvectors (loadings): Variable**	**PC 1**	**PC 2**	**PC 3**	**PC 4**	
ATMS	0.55865	−0.29664	−0.25654	−0.73082	
BRANCHES	0.406351	0.911573	−0.0448	−0.04366	
DEP__AC	0.473058	−0.1627	0.856515	0.126991	
LIFE_INS_	0.546813	−0.2336	−0.4456	0.669226	
**Ordinary correlations:**	**ATMS**	**BRANCHES**	**DEP__AC**	**LIFE_INS_**	
ATMS	1				
BRANCHES	0.292502	1			
DEP__AC	0.410679	0.264228	1		
LIFE_INS_	0.574689	0.307132	0.355631	1	

The purpose of this index is to simplify the results showing the effect of financial inclusion on economic growth. We have controlled the external factors by accounting for macroeconomic variables in our econometric analysis. These control variables include inflation (annual%) for consumer prices (INF), population growth rate (POP), trade percentage of GDP (TRADE), unemployment, education, corruption, and infrastructure (Mankiw, 2011; Kim et al., 2018). We used GDP per capita (GDP) as a proxy variable for economic growth and macroeconomic factors for dynamic panel regression to control financial inclusion, NPL, and economic growth. Hence, we set up the macroeconomic variables.

## Methodology

This study estimates the models using the robust standard errors proposed by Driscoll and Kraay (1998) for panel regressions with cross-sectional dependence. Specifically, we adopted the xtscc program developed by Hoechle (2007) that produces Driscoll and Kraay (1998) standard errors for linear panel models because they are not only heteroskedasticity consistent but also robust to very general forms of cross-sectional and temporal dependence (Le and Tran-Nam, 2018). Furthermore, the xtscc program works well with both balanced and unbalanced panels, which is the case in this study. It can also handle the missing values (Hoechle, 2007). For comparison and completeness, we estimate Driscoll and Kraay (1998) standard errors for coefficients using the pooled Ordinary Least Squares (OLS)/Weighted Least Squares, fixed-effects (within), and Generalized Least Squares random effects regressions. The Monte Carlo experiments show that, regardless of whether a panel is balanced or not, ignoring spatial correlation in panel regressions leads to overly optimistic (anti-conservative) standard error estimates, which is consistent with Driscoll and Kraay's original finding for small balanced panels. Although the standard errors of Driscoll and Kraay are modestly optimistic, their small sample features are much better than those of the alternative covariance estimators when the cross-sectional dependency is present.

Based on the objective of our study, we present the following models to explain:

Models 1 and 2. Impact of financial inclusion and NPLs on economic growth.

Models 3 and 4. Impact of NPLs and economic growth on financial inclusion.

Models 5 and 6. Impact of financial inclusion and economic growth on NPLs.

Model 1:


(1) $$\begin{array}{l} \text{LNGD}{\text{P}}_{\text{it}}=\alpha_{0}+\beta_{1}\ln\text{ NP}{\text{L}}_{\text{it}}+\beta_{2}\text{LnIn}{\text{f}}_{\text{it}}+\beta_{3}\ln\text{ trad}{\text{e}}_{\text{it}}+\beta_{4}\text{Po}{\text{p}}_{\text{it}}\\ \;+\beta_{5}\text{Edu}+\beta_{6}\ln\text{ C}+\beta_{7}\text{ROL}+\beta_{8}\ln\text{ INFRA}\\ \;+\beta_{9}\text{Unemp}+\varepsilon_{\text{it}} \end{array}$$

Model 2:


(2) $$\begin{array}{l} \text{LNGD}{\text{P}}_{\text{it}}=\alpha_{0}+\beta_{1}\text{Finde}{\text{x}}_{\text{it}}+\beta_{2}\text{LnIn}{\text{f}}_{\text{it}}+\beta_{3}\ln\text{ trad}{\text{e}}_{\text{it}}+\beta_{4}\text{Po}{\text{p}}_{\text{it}}\\ \;+\beta_{5}\text{Edu}+\beta_{6}\ln\text{ C}+\beta_{7}\text{ROL}+\beta_{8}\ln\;+\beta_{9}\text{Unemp}+\varepsilon_{\text{it}} \end{array}$$

Model 3:


(3) $$\begin{array}{l} \text{LnFinde}{\text{x}}_{\text{it}}=\alpha_{0}+\beta_{1}\text{NP}{\text{L}}_{\text{it}}+\beta_{2}\text{In}{\text{f}}_{\text{it}}+\beta_{3}\ln\text{ trad}{\text{e}}_{\text{it}}+\beta_{4}\text{Po}{\text{p}}_{\text{it}}\\ \;+\beta_{5}\text{Edu}+\beta_{6}\ln\text{ C}+\beta_{7}\text{ROL}+\beta_{8}\ln\text{ INFRA}\\ \;+\beta_{9}\text{Unemp}+\varepsilon_{\text{it}} \end{array}$$

Model 4:


(4) $$\begin{array}{l} \text{LnFinde}{\text{x}}_{\text{it}}=\alpha_{0}+\beta_{1}\text{GD}{\text{P}}_{\text{it}}+\beta_{2}\text{In}{\text{f}}_{\text{it}}+\beta_{3}\ln\text{ trad}{\text{e}}_{\text{it}}+\beta_{4}\text{Po}{\text{p}}_{\text{it}}\\ \;+\beta_{5}\text{Edu}+\beta_{6}\ln\text{ C}+\beta_{7}\text{ROL}+\beta_{8}\ln\text{ INFRA}\\ \;+\beta_{9}\text{Unemp}+\varepsilon_{\text{it}} \end{array}$$

Model 5:


(5) $$\begin{array}{l} \text{NP}{\text{L}}_{\text{it}}=\alpha_{0}+\beta_{1}\text{FINDE}{\text{X}}_{\text{it}}+\beta_{2}\text{LnIn}{\text{f}}_{\text{it}}+\beta_{3}\ln\text{ trad}{\text{e}}_{\text{it}}+\beta_{4}\text{Po}{\text{p}}_{\text{it}}\\ \;+\beta_{5}\text{Edu}+\beta_{6}\ln\text{ C}+\beta_{7}\text{ROL}+\beta_{8}\ln\text{ INFRA}\\ \;+\beta_{9}\text{Unemp}+\varepsilon_{\text{it}} \end{array}$$

Model 6:


(6) $$\begin{array}{l} \text{NP}{\text{L}}_{\text{it}}=\alpha_{0}+\beta_{1}\text{GD}{\text{P}}_{\text{it}}+\beta_{2}\text{LnIn}{\text{f}}_{\text{it}}+\beta_{3}\ln\text{ trad}{\text{e}}_{\text{it}}+\beta_{4}\text{Po}{\text{p}}_{\text{it}}\\ \;+\beta_{5}\text{Edu}+\beta_{6}\ln\text{ C}+\beta_{7}\text{ROL}+\beta_{8}\ln\text{ INFRA}\\ \;+\beta_{9}\text{Unemp}+\varepsilon_{\text{it}} \end{array}$$

where i represents 21 cross-sectional selected OECD countries, t is the period of the proxy measurement from 2004 to 2017, and ε_*it*_ is the error term. In models 1 and 2 specifications, the dependent variable is LnGDP as a proxy variable for economic growth; and in models 2 and 3, LnFindex is the proxy of financial inclusion variables. In models 3 and 4, NPL is the dependent variable that denotes NPLs. In all the models, Inf stands for inflation, trade is self-explanatory, Pop denotes the total population, Edu stands for education, C means Corruption, ROL shows Rule of Law, and INFRA stands for infrastructure in the OECD countries. Infrastructure is an index of three proxies namely energy use, fixed broadband, and phone usage.

Prior to panel regressions, we start our estimations from descriptive statistics followed by correlation, tests for multicollinearity, and heteroskedasticity.

### Descriptive Statistics

In this paper, we investigate the dynamic relations among financial inclusion, NPLs, and economic growth in the OECD countries over the period 2004–2017. Table 3 provides descriptive statistics related to our variables of interest and related macroeconomic variables.

**Table 3:** Descriptive statistics.

	**LnGDP**	**NPL**	**LNFindex**	**EDU**	**LnINF**	**LnC**	**LnINFRA**	**LnTrade**	**POP**	**ROL**	**Unemp**
Mean	4.434	4.347	2.727	0.989	2.504	1.794	3.270	1.930	0.424	1.100	7.412
Median	4.423	2.5	2.729	0.992	2.080	1.819	3.286	1.923	0.438	1.106	6.925
Maximum	4.961	42.39	3.263	1.058	15.430	1.986	3.965	2.247	2.530	2.100	27.47
Minimum	3.932	0.1	2.094	0.931	−1.735	1.462	2.814	1.378	−2.081	−0.667	1.96
Std. dev.	0.292	5.964	0.256	0.015	2.621	0.135	0.229	0.185	0.693	0.667	4.073
Observations	294	294	294	294	294	294	294	294	294	294	294

According to the descriptive statistics, the mean (median) values of LnGDP, LnNPL, and LnFindex are 4.43 (4.42), 4.34 (2.50), and 2.73 (2.73), respectively. At the same time, the statistics reveal that our sample countries have scattered values of NPLs indicated by the standard deviation of 5.96. This is also indicated by maximum (minimum) values, i.e., 44.39 (0.10). However, the LnFindex depicting financial inclusion has a mean (median) of 2.57 (2.57) with a standard deviation of 0.25. It means that our sample countries have similar indicators of financial inclusion, but the real issue is NPLs indicated in the discussion.

### Correlation

Next, the correlation is checked among the study variables. GDP is found positively correlated with financial inclusion and negatively correlated with NPLs. Similarly, NPLs are negatively correlated with financial inclusion. The results are shown in Table 4.

**Table 4:** Correlation.

**Variables**	**LNGDP**	**NPL**	**LNFI**	**EDU**	**INF**	**LNC**	**LNINFRA**	**LNT**	**POP**	**ROL**	**U**
LNGDP	1										
NPL	−0.183^***^	1									
LNFI	0.219^***^	−0.065	1								
EDU	0.361^***^	−3.973	0.114^**^	1							
INF	−0.408^***^	−0.114^**^	−0.090	−0.304^***^	1						
LNC	0.770^***^	−0.385^***^	0.319^***^	0.191^***^	−0.305^***^	1					
LNINFRA	0.684^***^	−0.230^***^	0.363^***^	0.323^***^	−0.156^***^	0.671^***^	1				
LNT	0.024	−0.079	−0.146^**^	0.136^**^	−0.066	0.205^***^	0.257^***^	1			
POP	0.212^***^	−0.405^***^	−0.069	0.051	0.213^***^	0.149^**^	0.132^**^	−0.202^***^	1		
ROL	0.754^***^	−0.355^***^	0.339^***^	0.201^***^	−0.295^***^	0.884^***^	0.693^***^	0.183^***^	0.069	1	
U	−0.332^***^	0.765^***^	0.011	−0.114^**^	−0.035	−0.379^***^	−0.357^***^	−0.043	−0.494^***^	−0.301^***^	1

*** *Significant value at 1% and*

** *significant value at 5%*.

### VIF Test for Multicollinearity

Next, this study analyzes the data set for multicollinearity. We use the variance inflation factor (VIF) for this purpose. The VIF measures how much the variance of an estimated regression coefficient increases if our predictors are correlated. Multicollinearity occurs when two or more predictors in the model are correlated and provide redundant information about the response. Multicollinearity was measured by VIF and tolerance. If the VIF value exceeds 4.0, or by tolerance <0.2, then there is a problem of multicollinearity (Hair et al., 2010). The results are shown in Table 5.

**Table 5:** VIF analysis.

	**GDP**		**FI**		**NPL**	
	**VIF**	**1/VIF**	**VIF**	**1/VIF**	**VIF**	**1/VIF**
GDP	–	–	4.32	0.231325	4.20	0.237871
lnFindex (Financial inclusion index)	1.39	0.718469	–	–	1.44	0.694443
NPL (non-performing loan)	3.03	0.330000	3.22	0.310187	–	–
ROL (rule of law)	5.52	0.181058	5.88	0.170085	5.38	0.185991
Lnc (corruption)	5.30	0.188619	5.77	0.173187	5.89	0.169789
Unemp (un employment)	3.26	0.306622	3.24	0.308533	1.60	0.626873
lnINFRA (infrastructure)	2.74	0.365243	2.68	0.372672	2.89	0.346210
Pop (population growth)	1.58	0.632845	1.62	0.617811	1.61	0.620050
InINF (inflation)	1.39	0.718185	1.51	0.663083	1.49	0.669839
lnTrade (Trade)	1.34	0.746802	1.27	0.787353	1.47	0.678395
Edu (education)	1.25	0.797772	1.31	0.766231	1.32	0.758754

### Test for Endogeneity

Moving forward, Table 6 confirms the application of fixed effects for our data set due to the existence of endogeneity in data. There exist arbitrarily distributed fixed individual impacts as shown in the results. The null hypothesis is rejected at 1% in favor of fixed effects. But the model cannot be estimated using the OLS techniques due to the existence of endogenous variables. The results are depicted in Table 6.

**Table 6:** Hausman test.

	**lnGDP**	**lnFI**	**NPL**
Chi-Sq. statistic	46.741950	24.204855	54.826368
Prob.	0.0000	0.0071	0.0000

### Test for Heteroskedasticity

Prior to estimation, the assumption of heteroskedasticity was tested using the Breusch-Pagan test. The results in Table 7 confirm the existence of heteroskedasticity at a 1% level.

**Table 7:** Heteroskedasticity.

**Breusch-Pagan**	**lnGDP**	**lnFI**	**NPL**
chi2(1)	162.03	35.64	5.10
Prob.	0.000	0.0000	0.0239

### Driscoll and Kraay Test for Fixed Effects

This study estimates the models using the robust standard errors proposed by Driscoll and Kraay (1998) for panel regressions with cross-sectional dependence. Specifically, we adopted the xtscc program developed by Hoechle (2007) which produces Driscoll and Kraay (1998) standard errors for linear panel models since they are not only heteroskedasticity consistent but also robust to very general forms of cross-sectional and temporal dependence (Le and Tran-Nam, 2018). The results are reported in Table 8.

**Table 8:** Estimation results: Driscoll-Kraay standard errors with fixed effect.

	**GDP**		**FI**		**NPL**	
	**Model-1**	**Model-2**	**Model-3**	**Model-4**	**Model-5**	**Model-6**
c	2.8431^***^ (0.3539)	2.6604^***^ (0.2738)	−0.2552 (0.5353)	−4.4733^***^ (0.6784)	−31.2873 (19.5481)	−28.27124 (21.2263)
RoL	0.0123 (0.0104)	0.0307^***^ (0.0068)	−0.0977^***^ (0.0237)	−0.1105^***^ (0 0.0104)	−3.6036^***^ (0.67698)	−2.9518^***^ (0.66519)
LnC	0.0874^***^ (0.0243)	0.1339^***^ (0.02407)	−0.0878^**^ (0.0441)	−0.2093^***^ (0.0522)	−5.9778^***^ (3.1637)	0.11429 (3.2584)
Unemp	−0.0075^***^ (0.0006)	−0.0067^***^ (0.00064)	−0.0015 (0.0010)	−0 0.0092 (0.0013)	1.0560^***^ (0.0869)	1.0876^***^ (0.1041)
lnInfra	0.1855^***^ (0.0239)	0.11582^***^ (0.0218)	0.5111^***^ (0 0.0637)	0.2564^**^ (0.1299)	7.3122^***^ (1.3696)	7.9118^***^ (1.7257)
Pop	0.0028 (0.0028)	0.0059 (0.0015)	−0.0043 (0.01224)	−0.0058 (0.0075)	−0.7268 (0.60227)	−0.5174 (0.4917)
LnInf	−0.0027^***^ (0.0009)	−0.0022^**^ (0.0010)	−0.0007^**^ (0.0003)	0.0027^**^ (0.0010)	−0.1574 (0.20313)	−0.29266 (0.2249)
lnT	0.1534^**^ (0.0603)	0.1630^**^ (0.0542)	0.0813^*^ (0.046)	0.1470^**^ (0.1000)	−0.67463 (0.5839)	−3.4772^***^ (0.63796)
Edu	0.5872^***^ (0.1244)	0.4065^***^ (0.0579)	1.4528^**^ (0.56415)	0.7040^***^ (0.2190)	−0.8633^***^ (15.9166)	16.5315^***^ (16.2773)
NPL	0.0018^***^ (0.00057)	–	−0.0020^**^ (0.00089)	–	4.5471^***^ (1.5192)	
lnFindex	–	0.1676^***^ (0.0124)	–	–		−2.5523^***^ (0.9258)
lnGDP	–	–	–	1.4373^***^ (0.2601)	−6.8751^***^ (1.2688)	

*,**,*** *Represent 10%, 5%, and 1% significance levels*.

Model 1 shows the effect of NPLs on economic growth. In contrast, inflation, population, trade, education, corruption, and the rule of law represent control variables that may affect the economic growth directly and indirectly. A 1% increase in NPLs increases 0.008% economic growth in the OECD countries. Hence, we reject the hypothesis H_5_. It implies that NPLs accelerate economic activities in this region of countries and result in increased economic growth. The results of Table 7 reveal that rule of law, corruption, infrastructure, trade, and education have a positive and significant impact on economic growth. It infers that economic growth is increased with the increase in these control variables. However, unemployment and inflation have a negative and significant link with economic growth as per theory. These two variables are unfavorable for economic development with different coefficients.

The results of model 2 in Table 3 reflect the significant positive effect of financial inclusion on economic growth. A 1% increase in financial inclusion causes a 0.167% increase in economic growth; therefore, we accept hypothesis H_1_. Our findings match with the results of Kim et al. (2018), who reported the same result for OIC countries. This result implies that when credit disbursement is increased in a country, economic activities are grown and the living standard of ordinary people improves. These results indicate an increase in the economic growth rate.

The third model presents the effect of NPLs on financial inclusion. There is a negative effect of NPLs on financial inclusion in the presence of control variables. A 1% rise in NPLs declines the financial inclusion level by 0.0020%; hence, we accept hypothesis H_4_. An increase in NPLs discourages the financial institutions from decreasing their credit disbursements. From the set of control variables, there is a negative impact of the rule of law, corruption, inflation, unemployment, and population on financial inclusion. Whereas, trade, education, and infrastructure increase the level of financial inclusion in the OECD countries.

Next, the results of model 4 reveal the positive but insignificant effect of economic growth on financial inclusion. It enables us to accept hypothesis H_2_. This finding state that the rural sector still lacks the financial access. The positive relationship between infrastructure and financial inclusion also endorses that weak infrastructure can be the leading cause of financial deprivation. Similarly, unemployment, population, and corruption also negatively impact the financial inclusion. Furthermore, trade, education, and inflation are positively related to financial inclusion, which has proved to be very helpful in the financial development of the OECD countries.

Model 5 reveals the effect of economic growth on NPLs. The results show that a 1% increase in GDP decreases the NPLs by 6.875% and rejects hypothesis H_6_. This relationship indicates that when living standards are better in a country, there are very few chances of misuse of credit. But it depends on the behavior of other control variables also. This finding is backed by the GDP growth rates of the OECD countries that GDP rates are increasing and NPL growth is declining.

Model 6, as shown in Table 8, presents the impact of financial inclusion on NPLs. The results show the adverse effects of financial inclusion on NPLs. A 1% increase in financial inclusion reduces the NPLs by 2.552%. This finding suggests that when people have more earning opportunities, they try to consume the loans as per their original purpose and there are fewer chances of NPLs. Control variables also suggest some exciting facts about NPLs. For example, rule of law, education, and trade reduce the NPLs, whereas inflation, corruption, and unemployment cause an increase in NPLs in the OECD countries.

## Discussion

In the recent era, the concerns regarding financial inclusion are rapidly growing. Scant literature explores the linkages between financial inclusion and economic growth. There is a need to study the linkages among financial inclusion, NPLs, and economic growth in the presence of macroeconomic variables for the OECD countries. The role of commercial banks in the OECD's banking sector has increasingly improved after reforms and open-up policies. The financial services of commercial banks have greatly enhanced the commodity resources of the banking sector as a whole. However, more financial services could lead to increased credit risks with the continuing growth of commercial banks, raising the question of non-performance loans. After the 2008 global financial crisis, the issue of non-performing commercial banks' loans became difficult to handle (Kauko, 2012). Against this backdrop, various commercial banks started seeking different control measures to alleviate the growth of NPLs, but these initiatives did not produce good results. According to the OECD market characteristics, it can be seen that local governments prefer a more national, rather than bank-specific, oriented emphasis on NPLs from commercial banks that are consistent with their regional economic development policies based on the overall local economy.

The results report the positive link between financial inclusion and economic growth for the OECD countries. The OECD countries need to strengthen the use of mobile banking in the countries. China is an example of such branchless banking. In far-flung rural areas, it is expensive to operate branch banking, therefore, digital finance can facilitate the users significantly. The government should introduce low-interest rates for low-income rural customers encouraging them to adopt a formal financial system. Financial literacy is also important for rural households to utilize formal banking channels and their use. The sample countries of our study include countries having diverse religious beliefs, literacy rates, monetary policies, and other macroeconomic factors. There are different schools of thought about the interest rate. These diverse factors signify the theoretical contribution of this study for financial inclusion.

## Conclusion

This study aims to understand the role of NPLs toward financial inclusion and economic growth. This study uses the Driscoll-Kraay standard errors with a fixed effect for a panel of 21 selected OECD countries. The results show a positive effect of NPLs on economic growth, but there is a negative effect of NPLs on financial inclusion in the presence of control variables. Furthermore, the findings of the panel dynamic regression technique reflect the positive and significant effect of financial inclusion on the economic growth of the OECD countries. The results indicate that rule of law, corruption, inflation, unemployment, and population hamper the level of financial inclusion, whereas, trade, education, and infrastructure behave otherwise. To cover the multicollinearity issue, we suggest establishing a financial inclusion index having a variety of factors explaining financial inclusion. Such an index will also be helpful to provide an accurate calculation of multilateral financial inclusion. This study is limited to the OECD countries and checks some well-known economic variables that affect financial inclusion and NPLs.

The study possesses a limitation in the availability of data. The data on financial inclusion is not available earlier than 2004. Similarly, data of all variables of all the OECD countries are also not available; therefore, only 23 countries were selected for analysis. Future studies can be conducted on the same topic by covering more panels and more updated data on study variables.

## Data Availability Statement

Publicly available datasets were analyzed in this study. The data that support the findings of this study are available in World Bank Indicators at https://data.worldbank.org/indicator, https://data.imf.org/?sk=E5DCAB7E-A5CA-4892-A6EA-598B5463A34C, and https://www.globalfoundationdd.org.

## Author Contributions

PZ conceptualized the idea of this paper and drafted the initial draft. MZ reviewed and improved this manuscript significantly. QZ supervised this study from beginning till end. SZ reviewed this article and recommended several policies for economic freedom. All authors contributed to the article and approved the submitted version.

## Funding

This work was funded by Philosophy and Social Science Planning Youth Project in Anhui Province Regional Studies of International Relations: Deconstruction and Reconstruction from the Perspective of Epistemology (Program Number: AHSKQ2021D39), Research on the Evolution of the ASEAN and AU Institution, a Key Project of the Humanities and Social Science Research Project of Anhui Province Colleges and Universities (Program Number: SK2020A0198), Anhui University of Science and Technology's Introduction of Talents Research Start-Up Fund ASEAN, AU Institution Evolution Research (Program Number: 13200382), and Hefei City 2022 Philosophy and Social Science Planning Youth Project Research on Three Governance Linkage in Rural Revitalization (Program Number: HFSKQN202250).

## Conflict of Interest

The authors declare that the research was conducted in the absence of any commercial or financial relationships that could be construed as a potential conflict of interest.

## Publisher's Note

All claims expressed in this article are solely those of the authors and do not necessarily represent those of their affiliated organizations, or those of the publisher, the editors and the reviewers. Any product that may be evaluated in this article, or claim that may be made by its manufacturer, is not guaranteed or endorsed by the publisher.
